# Rethinking real-time learning in debriefing: liminality, microgenesis, and semiotic borders

**DOI:** 10.1186/s41077-026-00436-9

**Published:** 2026-04-10

**Authors:** José Luis Medina, Gabriel Hervas, Isaac Calduch, Patricia Silva, Esther León

**Affiliations:** 1 Department of Didactics and Educational Organization, Faculty of Education, University of Barcelona, Barcelona, Spain; 2 Clinical Simulation Laboratory, Faculty of Medicine, University of Barcelona, Barcelona, Spain

## Abstract

We offer a new interpretive framework to analyze and explain learning processes in real time during discursive interactions in clinical simulation, especially in debriefing. First, we question the computational metaphor of the mind, where the mind is conceptualized as a closed system that processes symbols linearly, typical of traditional cognitivist learning approaches with profound practical implications over debriefing. This approach is insufficient to explain the complexity of learning in situated, uncertain, and dynamic contexts such as debriefing. During debriefing, participants do not simply remember data or assimilate external conceptual structures ready to be used, but rather undergo more complex processes. They experience discontinuities, oscillations, and ambiguities in their thinking, while negotiating tensions and facing moments of cognitive and emotional instability. To capture this dynamic, we need a richer, more flexible, and more responsive approach to lived experience.

Given these limitations, new interpretative proposals are emerging that seek to explain learning in debriefing by describing both the logic behind the configuration of the participants’ interpretive frames and the intra-subjective and intersubjective dynamics that modulate them in real time. The goal is to provide theoretical models that help us to elucidate a phenomenon that cannot be understood through linear schemes or unidirectional causalities, as it unfolds in the non-linear flow of brief temporal sequences loaded with meaning.

To this end, we present a new interpretive framework in which we have integrated three fundamental theoretical concepts: *liminality*, *microgenesis*, and *semiotic border*. Although these concepts come from different theoretical traditions, we have united them in a conceptual framework that allows us to understand how frames of reference are configured during debriefing, without reducing them to fixed or pre-established conceptual structures, preserving their dynamic, contingent, and situated nature. Each of these concepts addresses, from different theoretical traditions, how meanings are constituted under conditions of structural instability and on very short time scales.

Finally, we present empirical indicators that allow us to identify these complex and “elusive” phenomena in practice, providing guidance for supporting debriefers’ practice in noticing and interpreting learning, and a key methodological tool for research analysis as an emerging qualitative coding scheme.

## Introduction

Simulation-based education has become a fundamental pedagogical tool in the training of health professionals. In a context characterized by increasing healthcare complexity, simulation-based education allows students and professionals to rehearse procedures, make decisions, and deploy communication skills in a safe environment that is free of risks for both them and real patients. However, what gives simulation high didactic value is not only the possibility of repeating clinical actions in controlled conditions, but also the subsequent debriefing that makes it possible to transform the experience of the simulation into relevant professional knowledge. However, understanding what actually occurs at the mental, cognitive, and affective levels during debriefing remains yet to be fully developed in theory.

Contemporary post-simulation analysis tends to be oriented to identifying participants’ performance gaps and working with the participants to identify why those gaps occurred, by exploring the frames^1^ of reference that guided their performance [1]. As Rudolph, Simon, Raemer & Eppich point out ([2], p. 1011), “The debriefer works backward from an observed performance gap to discover what frames (assumptions, goals, knowledge base) drove the actions contributing to that gap.”

This approach emphasizes the need to access the often implicit^2^ cognitive schemes underlying participants’ clinical performance, through respectful and structured dialogue. The identification and exploration of these frameworks not only allow the participant to understand why they acted in a certain way, but also to work with the debriefer to construct new, more effective interpretive frames for the future. As noted by Rudolph et al. ([1], p.364):

“Learning occurs when instructor and trainee explore the frames-actions-results causal sequence in reverse. The instructor then explores with the trainee what frames and linked actions led to the actual results and then collaborates with the trainee in developing alternative frames and actions for the future”.

It is difficult to disagree with the ideas that underpin this approach to debriefing, as they are rooted in a reflective tradition that has had a powerful influence on the training of health professionals [3]. However, gaining a detailed and in-depth understanding of the learning processes that occur during debriefing remains a theoretical challenge.

The idea of reflexively exploring the mental frames that guide students’ actions has contributed significantly to transforming debriefing into a meaningful learning space, and not a mere stage for technical correction or a feedback exercise. However, precisely because of the high adherence to these ideas and in orderto advance our understanding of the learning processes during debriefing, we must examine carefully the scope and limits of this approach, recognizing its considerable value but facilitating new perspectives to complement and enrich what has already been developed. Thus, just as debriefing invites participants to review their own frames of reference, the theory and practice of debriefing also require constant review and updating to remain relevant in increasingly complex training and clinical contexts.

To better understand learning during debriefing, we focus on two related aspects: first, the assumed linear relationship among frames of meaning, actions, and results, and the depth at which participants’ frames have been analyzed so far.

### The dominant linear conception of learning in debriefing: tensions and limits

The proposal to understand learning as the result of retrospective analysis of the causal sequence between mental frames, actions, and results—as proposed in the debriefing model focused on guided reflection [1]—implies a linearity and transparency in cognitive processes that are difficult to verify in real practice [4]. As Schoenfeld et al. pointed out [5], when examined closely, learning is a non-linear^3^ process.

This vision, although innovative at the time, remains anchored in the assumptions of the classical cognitivist paradigm, which understands cognition as a logical, ordered, and sequential process [6]. According to this perspective, the subject constructs internal mental representations—such as schemas or frames—that guide their actions in the world. These actions, in turn, generate results that can be evaluated a posteriori in terms of their coherence with, or deviation from, a desired performance [7].

Learning, then, can be understood as a reflective and retrospective process aimed at identifying the frames (such as assumptions, goals, and knowledge bases) that guided the actions contributing to the performance gap [2], reinterpreting and refining these pre-existing mental models, and subsequently adjusting or replacing them with more appropriate ones.

This linear logic, heir to the computational metaphor that compares the brain to a computer, has profoundly marked not only theoretical models of learning since the mid-20th century [8, 9], but also their practical applications in contexts such as clinical simulation. The main impact of this logic on debriefing has been to assume that there is a causal, linear and, therefore, unidirectional relationship between a person’s frame of meanings and their actions. Under this approach, discrepancies between observed performance and the standard deemed correct are explained through the concepts, ideas, and assumptions embedded in the learner’s prior frame [2]. Accordingly, the debriefer’s role is to identify the learner’s mental models and guide them toward a conceptual transformation that more effectively aligns meanings, decisions, and behaviors [10, 11]. This approach assumes that once mental models are revised or corrected, changes in future behavior are facilitated.

However, these ideas, although intuitively clear and attractive in their simplicity, have been the subject of criticism from approaches and theories that integrate the interactional, biological, cognitive, phenomenological^4^, and social dimensions of human experience. These criticisms do not merely point out minor inadequacies of the model but rather call into question its basic epistemological assumptions.

The most widespread criticisms come from the perspective of situated cognition, which integrates cognitive, social, and neurobiological perspectives on learning to study how knowledge and learning emerge *through* the coordination of activity *within* its own context [6]. From this perspective, scholars reject the notion that we first perceive the world, then represent it internally through symbols, and finally act accordingly [12]. This sequence—perception, symbolic processing, action—implies a hierarchy that artificially separates knowledge from action, as if thinking and doing were separate moments, and not intertwined dimensions of the same situated practice [13, 14]. As early as 1896, John Dewey himself criticized the mechanistic vision of the “reflex arc” (the idea that action is simply a reaction to a stimulus) and proposed, instead, a more integrated conception of the organism as a functional unit, in which perception and action are part of the same dynamic and interactive circuit [15].

This approach points out that every thought and every action are tailored to their environment [16]. That is, they are situated because what a subject perceives, conceives their actions, and physically does develop together [17]. From this perspective, thinking and learning are phenomena similar to adopting and maintaining a bodily posture or riding a bicycle. In these situations, each change of posture or each act of pedaling is controlled not by intentional applications of prescriptive mental protocols learned and stored in memory ready for use, but by “re-coordinations” of immediately preceding postures, conceptualizations, and movements. The “sense” of posture is not previously stored in the brain but is, rather, the ability to create one posture from another, the ability to establish a relationship [18]. Similarly, when during debriefing participants think about and produce descriptions of the processes that occurred during the simulation phase, make conjectures, or interpret what the debriefer’s statements mean, each moment of that process is not controlled by applying rules and ideas previously stored in memory, but by re-coordinating their immediately preceding thoughts and ideas. Any human action is at least partially improvised from the direct and dynamic coupling^5^ of perception, conception, and conceptualization [19]. This is a dynamic process that is not mediated by previously stored descriptions of concepts, rules, laws, or procedures [20].

In the more specific field of cognitive neuroscience, Skarda and Freeman [21] demonstrated that significant patterns of brain activity are self-organizing, emergent, and non-representational. For example, perception is a constructive process that depends on intrinsic stimulus information (stored as conceptual meaning) and, above all, on the organism’s neurobiological structure that processes and categorizes inputs. That is, concepts are grounded in neural circuits distributed in cortical regions involved in perception and action; perception, action, and cognition therefore rely largely on the same neural substrate. In this regard, Gerald Edelman^6^ [33] proposed that consciousness and behavior arise from selective and recursive processes in complex neural networks, without a fixed correspondence between stimulus, representation, and response. These pieces of evidence question the alleged causal and linear relationship “frame-action-result” and support the idea that the processes of perceiving, describing, conceiving, understanding, and acting unfold together and shape each other [12].

The idea that knowledge is stored available to be applied to situations does not resist neurobiological examination [22]. On the contrary, research in neuroscience and phenomenology have shown that the processes of perception, categorization, and comprehension are not supported by pre-formed symbolic structures, but emerge from a dynamic coupling between the organism and its environment [23]. That is, there is no mind that represents the world from outside it, but an embodied, corporeal, situated cognition that generates meaning in real time from ongoing interaction [24]. These ideas are aligned with those of Maturana and Varela [25] and Dreyfus and Dreyfus [26] who contributed to dismantling this linear model showing that action is not a simple execution that takes place after comprehension, but rather is constitutive of it.

From this perspective, learning cannot be reduced to updating or modifying internal cognitive structures. It is an emergent, dynamic, and contextual process in which understanding occurs within the action itself, rather than preceding it or being enacted from the outside [27]. Learning, understanding, and comprehension always occur in an emergent way in interactive contexts and not as the assimilation of external predefined conceptual structures that transform those that the subject possesses [28].

Finally, one of the most robust challenges to the idea that in debriefing we can causally reconstruct the frame-action-result sequence is the fact that this sequence simply does not exist as such in the lived experience of the participants [29]. In practice, what guides an action is not a set of stored rules, but a situated sensitivity that articulates understanding and action inseparably [30]. When seeking to investigate the causes of the gap between observed and expected performance, there is a risk of focusing attention on the conceptual contents that the participant “had in mind” during the simulation without attending to the dynamic process that generated them. But without understanding that dynamic—irreducible to the sequential logic of input, processing, and output—any attempt at feedback will be superficial and scarcely effective. What is urgent, then, is not only to identify the contents of the mental frame, but to understand how that frame was configured in real time, in a concrete practice, crosscut by specific conditions of interaction, corporality, emotion, and meaning [23].

In short, this dynamic approach to the learning process postulates that meaning is conceptualized in in real situations of use [31]. The situated cognition approach seriously questions the existence of stable mental representations of concepts—that is, the existence of a genuine conceptual structure (a “frame”)—at least in the degree of permanence and stability proposed by some cognitivist approaches to learning [20].

This non-linear, emergent understanding of cognition and learning has important consequences for our understanding of debriefing. If the processes of perception, conceptualization, and action are deployed in real time, simultaneously and interdependently, the attempt to retrospectively isolate a “failure” and attribute it to an existing mental frame activated during the simulation phase is problematic. The debriefer’s task, then, cannot be limited merely to a diachronic exploration with participants of the schemas and assumptions they activated during the simulation phase and it should also involve collaborating in the dialogical reconstruction of meanings that emerge from the ongoing interaction [28]. In this interaction, the questions, the answers, their correlation in the learning of the participants, and the internal processes of perception, categorization, and conceptualization that both they and the debriefer put into play, are not discrete entities, previously elaborated and stored in the brain and activated at a certain time to create interpretations, meanings, and answers. On the contrary, what we often see in debriefing as the “causal” result of mental activity (concepts, ideas, inferences) are reorganizations of experience (present and past) and orientations for the activity that is underway. For example, when during debriefing a participant asks the debriefer a question, this interaction is not a process in which a sequence of linear and causal relationships^7^ between three discrete entities can be identified: (a) participant question (stimulus), (b) inference (debriefer thinking), and (c) debriefer answer (response). The question does not act as a prior stimulus to which the debriefer *subsequently* reacts and mediated by a deliberation. Separating perception, categorization, and conceptualization as discrete phenomena and establishing a linear and causal sequence between them is incorrect [21]. While the debriefer listens to the question—and not afterward—what they perceive as relevant and what they believe needs to be understood are constructed in the present moment, simultaneously with the development of a particular understanding of what the participant wants to say. While the debriefer consciously listens to the participant, in their brain, a series of couplings and coordinations between their areas of perception, categorization, and conceptualization occur unconsciously. At a first micro-instant, not only is the answer uncertain, but the question (the stimulus) is also uncertain. One is uncertain, only to the extent that the other is [32]. They are correlative, and they are deployed simultaneously. The meaning of the question is something to be discovered; it must be constructed and, simultaneously, the answer, through this very same process, is also developed. In reality and as Dewey maintained on the reflex arc, stimulus and response are parts of a single coordination, and they obtain their meaning purely from the role played in maintaining or reconstituting that coordination [15]. As Edelman [33] has shown, perceptual and conceptual areas of the brain may be anatomically isolated but are functionally inseparable.

This change of perspective implies that debriefing, rather than being based on a linear, retrospective, and diachronic logic, which seeks to “reverse engineer” the simulated experience, as if it were possible to decompose it into causes and effects, could broaden its focus to include more situated and emerging forms of analysis by recognizing that comprehension occurs in the very act of narrating, conversing, and interpreting, and that meanings are not retrieved intact from memory, but are configured in real time, in an intersubjective space [6].

Consequently, debriefing becomes an inherently situated, open, interpretive, and non-linear practice, as close to a phenomenological conversation as to a technical analysis. This does not mean renouncing rigor or the intended pedagogical objectives but rethinking the way in which those objectives are constructed: not only as predefined goals to be achieved, but also as shared discoveries that emerge from an experience that is lived and narrated jointly [34].

### A new perspective on learning processes in debriefing

From these criticisms, new interpretive proposals have appeared that try to explain the learning processes that are deployed during debriefing, describing both the logic through which participants’ interpretive frameworks are configured, and the intra-subjective and intersubjective^8^ dynamics that modulate them in real time [4, 35]. The goal is to provide frameworks of interpretation that help us to grasp and elucidate a phenomenon that cannot be understood through linear schemas or unidirectional causalities [22] and that becomes the non-linear flow of brief temporal sequences loaded with meaning [36].

In the following, we present a new interpretive proposal in which we articulate three theoretical notions: *liminality*, *microgenesis*, and *semiotic border*. Although these emerge from different theoretical traditions, we have integrated them into a conceptual framework aimed at elucidating the dynamic process of configuring the frames of meanings that occur during debriefing, without reducing them to pre-established conceptual contents. Integrating these perspectives can help us address the complexity inherent in the meaning-making processes that characterize debriefing, preserving its dynamic, contingent, and situated nature. Each of these concepts addresses, from different theoretical traditions, how meanings are constituted under conditions of structural instability and on very short time scales.

### Liminality

The notion of *liminality* was developed in the field of social anthropology to describe the transition that people experience during rites of passage [37, 38]. Van Gennep [37] sought to understand the rites of passage through a triadic sequential structure (separation, transition, and incorporation), considering that usually the transition is not an immediate or sudden action. The liminal stage must be conceived as an interphase space between what is ceasing to be (separation) and what is beginning to be (incorporation). The term *liminality* comes from the Latin *limen*, which means “threshold.” This notion has acquired remarkable relevance, particularly in the social sciences, education, health, and organizational studies, where it has been used to account for processes in which subjects move between knowledge structures, identity states, or social roles without having yet reached a new stabilized order.

In the context of learning during discursive interactions in debriefing, liminality can be understood as a space of oscillatory ambiguity, differentiated from the previous and subsequent mental states with respect to a given act of learning and located in their interstices. Between the margins of these two moments (preliminal and postliminal) there is a space that links them, giving rise to a process of conceptual “re-coordination” between an already incorporated structure of initial significance and a new structure of emerging significance [28]. It is an interphase space between what is ceasing to be (no longer) and what is beginning to be (not yet): a logic of the “betwixt and between” [38].

Meyer and Land [39] argued that liminality can be a useful metaphor for better understanding the conceptual and ontological transformations and difficulties that students experience during their learning process. These authors define it as “a ‘liquid’ space, simultaneously transforming and being transformed by the learner as he or she moves through it” ([39], p. 380) and characterize it as a transformative state that takes existing certainties and makes them problematic and fluid [40].

Liminality implies a suspension of previous frames of reference and an exposure to structural and symbolic instability. Land, Rattray, and Vivian [41] define it as a “liminal learning space,” in which subjects face ideas that destabilize their previous way of knowing and place them in an uncertain territory where they have not yet integrated a new way of understanding. In this suspension, identity becomes transitory, and thought moves between the familiar and the unknown, which generates an oscillation between moments of regression and transformation.

The student who crosses the liminal space faces a series of epistemological and ontological obstacles, derived from the cognitive disturbances they experience during passage through that space [42] These obstacles frequently produce in the student a state of “suspension in which understanding approaches a form of mimicry or lack of authenticity” [40]. In this sense, students often adopt vocabularies and behaviors that they perceive as required, without understanding them and without a real capacity to assimilate and apply them. However, mimicry should not be understood as superficial, rote learning, but as the result of a genuine effort at comprehension, and even a preliminary step to deeper understanding. This causes, on many occasions, the student to experience an oscillatory process, in which they have the intermittent sensation of having understood something followed by the realization that their understanding has faded. During this passage through liminal space, the student must be able to let go of previous positions, which may give rise to a sense of loss, while they begin to appropriate the new ones. This conceptual integration and reconstitution of subjectivity occurs in an oscillating and non-linear way [43]. In this sense, liminality has been described as an emotionally intense and cognitively unstable experience. According to Piro and O’Callaghan [4], it is a state of “constructive destabilization,” in which individuals are forced to question their own certainties, exposing themselves to an experience of self-estrangement that can give rise to a subjective reworking. In this sense, passage through liminal space is not only cognitive, but also existential and affective [44]. This emotional dimension of the liminal learning process is not accessory but rather is its very condition [44]. Anxiety, uncertainty, enthusiasm, and/or shame are not difficulties or “deficits” attributable to the participants but are an integral part of the liminal transition. The feeling of “not knowing yet” or “no longer understanding as before” is constitutive of the liminal learning process.

The concept of liminality is particularly relevant because it will allow us to focus our attention on the dynamic transition that occurs in interactive learning processes during debriefing and not so much on the previous or subsequent states. We know that the discursive interactions between participants and debriefer have the potential to influence their trajectories within the liminal space and, therefore, their learning processes [4]. But we don’t know what the characteristics of that dynamic are.

Liminality allows us to conceive learning during debriefing not as a linear and incremental process of accumulation of concepts and/or procedures, but as a passage through states of conceptual and, sometimes, ontological “destructuring” and reconfiguration [44]. This approach recognizes that learning can legitimately involve disorientation, resistance, and even regression, to the extent that participants negotiate meanings in uncertain territories [43] and offers an interpretive framework that can help us understand the course of thought of the participants during these core moments on which their learning depends.

From this perspective, learning necessarily involves a process of destructuring: The subject must abandon, at least partially, some of their previous mental frames to open up to new ways of thinking, feeling, and acting. This transition is, by definition, unstable. Learning during debriefing does not follow a linear path or guarantee immediate understanding. It is a fluctuating, irregular process, full of regressions and partial advances that can generate anxiety, confusion, or even rejection. However, this “crisis” is constitutive of meaningful learning, since it allows the reconfiguration of meanings. Understanding learning as a liminal experience implies recognizing its discontinuous, uncertain and, often, painful character, but also its transformative power. Liminality favors deeper learning, since it destabilizes the known and allows it to be rearranged with new meanings [40].

### Empirical manifestations of liminality

Now, how can we identify these liminal learning processes during debriefing? How does the liminal learning process manifest itself during the discursive interactions that occur during it?

Based on a detailed and in-depth conceptual analysis of the notion of liminality, we have deductively identified a series of empirical indicators that are now in the process of validating and expanding inductively based on the empirical evidence in our ongoing research. These indicators are listed in Table 1.

**Table 1:** Empirical indicators of liminality

Dimension	Indicator	Operational definition
1. Non-linear processual dynamics	Non-linear dynamics	Discontinuities, advances and setbacks, and multidirectional movements in the participant’s discourse and learning process.
	Oscillation between certainty and doubt	Alternation between moments of apparent understanding and moments of confusion or conceptual insecurity.
	Non-linear conceptual transitions	Apparent regressions or detours followed by more complex or better-articulated reformulations
2. Epistemic rupture and destabilization	Experience of rupture or disturbance	Impossibility or difficulty of continuing to rely on a given idea or concept, often accompanied by feelings of disorientation, paralysis, or alienation.
	Suspension of certainty	Destabilization or interruption of knowledge previously taken as firm or self-evident.
	Collapse of previous competence	Recognition that previously effective knowledge or strategies no longer suffice to address the current situation.
3. Cognitive tension and ambiguity	Cognitive dissonance	Psychological discomfort resulting from the simultaneous maintenance of incompatible concepts, values, or interpretations. It is a notoriously devaluing, disabling, and difficult mental state to cope with
	Cognitive ambiguity	Co-presence of doubts, contradictions, or multiple interpretations without clear prioritization or resolution.
	Being stuck	Perception of being trapped and/or unable to move forward due to tension between competing conceptual structures.
	Cognitive regression	Return to earlier, less elaborated conceptualizations as a defensive response to uncertainty or ambiguity (search of certainty).
4. Conceptual and identity transformation	Transformation of the frame of reference	Changes in the way a situation is interpreted or justified.
	Instability in identity or concepts	Uncertainty regarding one’s role, knowledge, or conceptual positioning.
5. Affective manifestations of the process	Disruptive or intense affect	Heightened emotional intensity (e.g., anxiety, frustration, enthusiasm, confusion, and blockage) linked to the learning process.
	Emotional ambivalence	Coexistence of opposing emotional evaluations of the same experience.
	Affective volatility	Heightened emotional reactivity and susceptibility to being affected by the conversation.
6. Discursive coping strategies	Defensive reaction	Discursive strategies that externalize uncertainty or anxiety by discrediting new knowledge and avoiding self-questioning.
	Mimetic use of professional language	Formal use of technical terminology without evidence of conceptual understanding or justification.
	Liminal language	Use of ambiguous or indeterminate expressions that signal a transitional or in-between conceptual position.

Research in health science education and in simulation has studied liminality in a *molar* way [45–50]. In this work we propose a novel *molecular* and *microanalytical* approach to the key moments of this transition, in which the processes of differentiation and conceptual change (read: learning) are particularly intense. We propose that those turbulent micro-instants in which “something” is in the process of becoming are when we should turn our attention during debriefing.

This fine-grained approach should be applied to both the temporal and conceptual scales. Since very subtle distinctions in meaning are relevant and, therefore, should be traced, an appropriate level of “conceptual resolution” and analytical depth is necessary. We can achieve this using the concepts of *microgenesis* and *semiotic border*.

### Microgenesis

In developmental psychology, the term *microgenesis* was introduced by Heinz Werner [51] to indicate the “genetic” character of immediate experience and, more generally, to describe the dynamic structure of any psychological process.

The concept of microgenesis refers to the development on a short timescale of a perception, a thought, an idea, or an expression [51]. According to Rosenthal [52], it designates the emergence of immediate experience through a genetic dynamic of “differentiation” and “development.” Every thought process, says the author, is a microgenetic process that is constructed from an incipient background, which becomes structured through distinctions that emerge in the very course of cognitive activity. That which is given to consciousness in the form of an idea, a judgment, or comprehension is the result of multiple previous recursive moments of internal differentiation. But also, every thought or idea that emerges does so through a developmental process. Just as every biological organism follows its ontogenetic path, every immediate experience follows its path of microgenesis or *microdevelopment*, but on the scale of the present time ([52], p. 86]). These processes are characterized by progressive differentiation and development, ranging from more vague and undifferentiated stages to more defined and stable stages, which are those that are usually presented reflexively in consciousness ([53], p. 312).

Microgenesis occurs on an extremely short time scale, which makes it possible to observe the emergence and organization of a mental or cognitive activity in the instant in which it occurs [54]. The concept of microgenesis thus allows us to analyze learning as a series of microtransformations that are not always visible in the final result, but which are key to understanding the process of knowledge construction.

The idea of differentiation is the core of the orthogenetic principle formulated by Heinz Werner “wherever development occurs, it proceeds from a state of relative lack of differentiation to a state of increasing differentiation, articulation, and hierarchical integration” ([55], p. 866). Although this principle was formulated for the study of psychological and biological development, its extrapolation to the field of conceptual learning helps us explain how participants move, during debriefing, from vague or generalized formulations to more precise, hierarchical, and integrated conceptual distinctions. Microgenesis, in this context, allows us to capture that internal movement, moment by moment, in which concepts are deployed, reconfigured, or enriched. We observe the orthogenetic principle in action when, during debriefing we watch how a vague notion (for example, “effective communication”) is transformed into an articulated set of distinctions, nuances, and concrete practices.

Thus, microgenesis describes the immediate temporal development of emergent meaning, showing how an initial differentiation evolves over the course of interaction during debriefing. This evolution can follow different trajectories in immediate time: emergent meaning may progressively stabilize into a new conceptual structure, remain in a state of prolonged ambiguity, dissolve, or collapse back into prior formulations. Such regressions should not be interpreted as setbacks or involutions in a negative sense, but rather as necessary temporary returns to more familiar and less differentiated ideas that fulfill structuring and/or defensive functions [56].

In summary, during debriefing, concepts emerge by progressive differentiation from previous conceptual frames, not through direct application. What the participant “understands” is in a state of genesis: It is formed as they speak, listen, compare, and reflect. An idea or a clinical decision do not appear already developed, complete, and ready for use, but are shaped through progressive differentiation, anticipations, internal tensions, and transient syntheses.

### Empirical manifestations of microgenesis

The fact that microgenetic processes occur on very fine time scales makes it difficult to observe how participants make meanings, adjust their understandings, and reorganize their mental schemas. Table 2 presents a series of empirical indicators that we have found in our research, which can help identify the microgenetic processes that occur during debriefing.

**Table 2:** Empirical indicators of microgénesis

Dimension	Indicator	Operational definition
1. Non-linear evolution dynamics	Progressive reformulation	Successive changes in how an idea is expressed, indicating ongoing conceptual evolution and increasing understanding.
	Repetition with variation	Successive rehearsals of meaning through the repetition of ideas with small but meaningful modifications.
	Verbal rehearsals	Production of incomplete, self-corrected, or revised utterances that display the process of meaning construction in action.
2. Conceptual differentiation and precision	Refinement of the conceptual structure	Gradual development from general or imprecise formulations toward more coherent, articulated, and clearly delimited concepts.
	Increase in conceptual differentiation within the same semantic field	Introduction of nuances, subcategories, contrasts, or counterexamples within the semantic field.
	Resignification of concepts	Use of terms that are new to the participant or redefinition of familiar terms to express a transformed understanding.
3. Relational and integrative conceptual development	Progressive and relational articulation of ideas	Linking earlier formulations with new ones in a relational, non-additive manner that restructures understanding.
	Emergence of hierarchical or integrated structures	Development of conceptual organizations with multiple levels of differentiation and integrative reconciliation.
4. Conceptual stabilization and consolidation	Consolidation of a distinction	Stabilization of a newly acquired conceptual differentiation that is subsequently used consistently to guide interpretation or action.

In learning microgenesis, the primary act of differentiation, the turning point where conceptual novelty emerges, is produced by so-called *semiotic borders*. It is within these mental devices that the emergence of new meanings, essential for the learning process during debriefing, is made possible.

### Semiotic borders

The idea that the first operation of psychic life is the emergence of consciousness from the segmentation, differentiation, and stabilization of a flow of thought into discrete units, called ideas, is not new [57]. Herbst [58] suggested that the genesis of logic and behavior lies in the production of a distinction in a stream of indistinguishable events. Similarly, Bateson ([27], p. 92) pointed out that every mental process is activated by the production of a difference understood as a transformed and elaborated version of the difference that preceded it. For his part, Greimas [59] revealed how the fundamental structure of signification is rooted in a primary process of differentiation. This act of differentiation is a semiotic process^9^ that occurs with the appearance of the sign, that is, a discrete form that emerges from an undifferentiated continuum. However, that act of distinction does not create separate ideas or notions but rather generates a relational system. Drawing a distinction creates a boundary that, simultaneously, divides and unites two notions where previously there was a single undifferentiated instance [60].

To achieve this differentiation of meanings, to be able to perceive that they are different, it is necessary to establish boundaries between them. The mental device that produces these limits and distinctions is the *semiotic border* [61]. Therefore, all signification begins with a primary distinction, that is, with the act of drawing a semiotic border [62].

The term semiotic border was introduced by Juri Lotman, in the context of his theory of the *semiosphere*^10^ to designate the areas of contact between different systems of meaning, that is, different semiotic systems. These borders, like geographical ones, not only act as dividers, but also allow the exchange or transformation of meanings. They are active places of the production of meaning, not simply passive barriers.

In general, we can assume that a border is a mechanism that promotes the production of a discontinuity. This means extracting from an undifferentiated and continuous background a discrete form, a sign, which makes it possible to create at least one difference. The first differentiation is precisely the creation of a border, which helps to build and delimit a form^11^ ([62], p. 3).

**Fig. 1:**
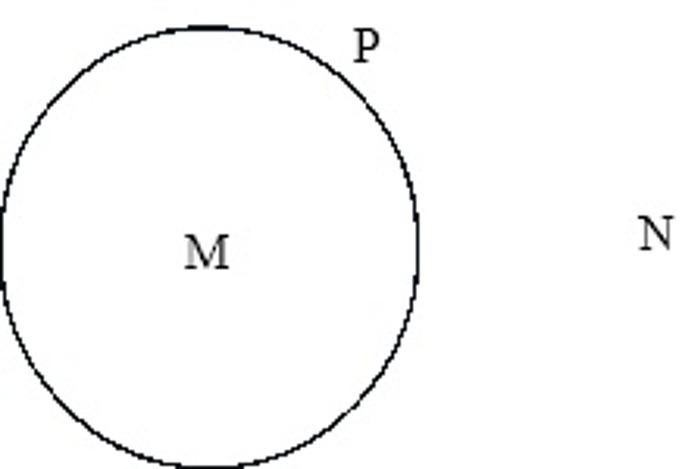
Triad of components in a primary distinction. M: the content that is internal to the distinction. N: the content that is external to the distinction. P: the border or limit that separates and at the same time connects and defines both

How does that primary distinction come about? David Herbst [58] developed a logical system that sought to explain “the relationship between our intentions and the conceptual and rational forms in terms of which we perceive and respond to ourselves and the environment”^12^ ([58], p. 84). His first step was to identify the primary operation—the genetic basis of logic and behavior. According to him, this primary operation is the production of a “distinction” within an undifferentiated field of events. By making this distinction, a structure of three components is simultaneously generated (Fig. 1):

The first axiom of Herbst’s logic indicates that “the primary conceptual unit is given as a triad of distinguishable undefined components, which are definable in terms of one another” ([58], p. 90). It is easy to see that the only operation necessary to create the triad in Fig. 1 is to draw the border (the distinction) “P”. Before this operation, in an undifferentiated state, none of the elements existed. Once we draw the circumference “P,” we immediately obtain an internal (“M”) and an external (“N”) element. This structure cannot exist partially. By eliminating the limit “P,” it is no longer possible to distinguish between the inside and the outside. The same result is obtained if the interior or exterior is removed, since then the other two components of the triad also disappear ([58], p. 89). Therefore, in this triad, all parts arise at the same time and disappear simultaneously by eliminating the border. The “outside” is the inevitable context of the “inside,” and the border both separates and unifies them [63]. The emergence of any form of distinction simultaneously cogenerates this triad as a whole in which the elements are codefined by their relationships.

Note that this triadic structure is not simply a descriptive model, but expresses a relational ontology, in which concepts do not pre-exist in isolation from their relationships, but emerge from them. Meaning, in this view, is a relational phenomenon that emerges through structural interconnections, not from the atomicity of discrete units [61]. Therefore, for new meanings to emerge, the function of separation and distinction between different concepts is not sufficient. Semiotic boundaries, while establishing these primary differentiations, relate and connect the concepts that are being differentiated through interpretation and translation processes [64].

We thus arrive at one of the fundamental characteristics of semiotic borders: their ambivalent and apparently paradoxical nature. Semiotic borders separate while unifying [65]. This paradox is easy to understand if we think of the two sides of a border between states. On one side, the border touches and is part of a state. On the other, it touches and is part of the other state. But the border is not composed only of those two sides; there is also a third space located between the two sides that touches the states. According to Nail [66], this interstitial space produces a phenomenon of inclusive disjunction, a diffuse zone very similar to a liminal space. This ambivalence is what helps us to understand the intrinsic dynamics of continuity/discontinuity that occur between conceptual spaces [67].

The construction of a semiotic border and its crossing involve constitutively temporary movements and dynamics. We understand the semiotic border not only as the fixed segmentation of a field and/or the definition of its parts, but also as a dialectical process of stabilization and transformation of notions and meanings over time [68]. These temporal dimension is not as a mere linear succession of events, but the emergence of a complex intertwining between available meanings that are being transformed and others that begin to emerge [69].

The crossing of a semiotic border, however, can have different speeds and durations [70]; a new meaning can emerge suddenly and abruptly—in the manner of a Gestalt insight—or through more gradual processes of discretization over a longer period [68].

The peculiar dynamics and the semiotic structure of the borders generate a turbulent space, which is characterized by instability, by a diffuse spatiotemporal distinction, and by ambiguities in the semantic and syntactic processes of construction of meaning that generate a strong affective charge [61]. The semiotic border is a zone of passage that is not only cognitive, but also affective, marked by uncertainty, openness, and the potential for rearticulation. This condition of indeterminacy has also an inherently paradoxical character. On the one hand, it can produce blockage, anxiety, or regression, to the point that not all subjects successfully cross the semiotic border [41] and might become trapped in a sort of “limbo” of persistent ambiguity. On the other hand, the semiotic border is also a source of creative potential, since by breaking with stabilized structures it allows new meanings to emerge [70].

In short, from this semiotic perspective, learning in real time during debriefing can be understood as a process in which the subject produces semiotic borders during their encounter with new knowledge. It is not a simple assimilation of previously defined contents, but a generative act in which the subject draws borders that configure significant distinctions within a part of the initially undifferentiated experiential and professional field. This operation implies a discontinuity with previous forms of meaning, giving rise to a space of ambiguity and paradox in which the known and the emerging coexist unstably. In that border space, the subject must face the simultaneity of continuity and discontinuity, of stability and change, through an active process of semiotic reorganization that allows them to integrate or re-signify what is happening.

Thus, debriefing is not merely a retrospective reflective exercise, but a “constitutive” moment in which the subject is placed in a zone of conceptual and affective passage, where uncertainty and ambivalence are not obstacles to learning, but rather necessary conditions for the emergence of new meanings.

How can we identify the appearance of these semiotic borders in the discourse of debriefing participants? How do these elusive phenomena that occur in the subjectivity of the participants manifest themselves?

### Empirical manifestations of semiotic borders

Table 3 shows some empirical indicators of semiotic borders that can help us identify these elusive phenomena that are fundamental to understanding the learning processes that occur during discursive interactions in debriefing.

**Table 3:** Empirical indicators of semiotic borders

Dimension	Indicator	Operational definition
1. Conceptual differentiation and precision	Explicit conceptual distinctions	Conscious and explicit signaling of conceptual differences that were not previously recognized or articulated.
	Emergence of distinctions	Spontaneous appearance of conceptual differences that were not previously present in the participant’s discourse.
	Production of contrasts	Generation of distinctions that delimit categories, meanings, or conceptual positions.
	Contrast or comparison between notions	Deliberate differentiation between similar, opposing, or complementary concepts in order to delimit meaning.
2. Relational and integrative conceptual development	Inclusive disjunction	Coexistence of divergent explanations or perspectives that are actively negotiated and partially integrated rather than mutually excluded.
	Detection of a triadic relational structure (inside–border–outside)	Emergence of a structured conceptual field organized around inclusion, exclusion, and boundary positions.
3. Conceptual categorization and reorganization	Establishing a new category	Introduction of a new term or label that organizes previously undifferentiated phenomena into a coherent category.
	Making the tacit background explicit	Explicit verbalization of previously implicit assumptions, values, or background knowledge guiding understanding or action.
	Recognition of ruptures, discontinuities, and interpretative limits	Identification of moments where prior knowledge or interpretive frameworks are no longer sufficient (“This changes what I thought”).
4. Cognitive tension and ambiguity	Conceptual ambivalence	Verbal expressions in which two ideas coexist simultaneously, often spanning multiple or antithetical categories.
5. Discursive meaning-making strategies	Production of translations, analogies, or metaphors	Discursive attempts to translate meaning across closely related conceptual frames through analogy, metaphor, or reformulation.

## Conclusions

From a theoretical and conceptual standpoint, the integration of the notions of liminality, microgenesis, and semiotic boundary makes it possible to construct a multilevel theoretical framework to describe and explain the learning processes that emerge during debriefing. From a formal and functional perspective, these notions, rather than operating as juxtaposed concepts, are articulated as a conceptual ecology in which each fulfills a specific yet interdependent analytical function. Although these notions can be conceptually isolated, they should be considered functionally inseparable: they co-organize and mutually influence one another while remaining coherent in themselves. Articulated together, they form a structure of three nested levels of increasing analytical depth, organized according to their explanatory function and temporal scale.

The integration of these notions makes it possible to understand learning during debriefing as an emergent, non-linear, and situated phenomenon that unfolds simultaneously across different analytical levels and temporal scales and conforms a coherent and robust conceptual framework for analyzing how participants produce meaningful conceptual differences in real time, in situations in which meaning has not yet stabilized.

Within this framework, liminality corresponds to the first analytical level and the broadest temporal scale, as it defines the general context of transition: an extended, turbulent, and ambiguous space-time of cognitive—and at times identity—transformation, in which participants’ prior frameworks are suspended without a new stable conceptual structure having yet been consolidated [46]. Within this space-time, microgenesis emerges at an intermediate analytical level and on a shorter temporal scale, corresponding to the succession of micro-moments of transformation through which a conceptual distinction progressively develops and comes into consciousness in the form of an idea or concept [52]. At the deepest analytical level of this process, semiotic borders appear on a very fine temporal scale, as punctual micro-instances in which the initial emergence of a meaningful difference occurs, marking the inflection point at which what is relevant is first differentiated from a previously undifferentiated zone [62].

Together, these constructs make it possible to shift attention away from models of learning based on the application of pre-existing mental representations toward a more phenomenological, situated, and dynamic understanding centered on the emergence of meaning through interaction. This shift is not only conceptual but also methodological, as it invites the analysis of debriefing interactions as scenarios of thinking-in-action, in which learning is not simply the result of applying knowledge, but of producing it in real time through lived, shared, and collectively reflected experience.

As we have argued, just as debriefing invites participants to critically examine their own frames of reference, assumptions, and habitual ways of making sense of experience, the theory and practice of debriefing themselves require a similar reflexive stance. This reflexivity should not be understood as a purely theoretical exercise, but as an ongoing professional commitment for educators to interrogate how debriefing models are enacted, adapted, and sometimes constrained within specific educational, organizational, and clinical contexts. For educators, this implies continuous attention to their own assumptions about learning, performance, and learner agency, as well as openness to revising established debriefing practices in light of emerging evidence and new theoretical developments. From this perspective, concepts such as liminality, microgenesis, and semiotic border offer valuable analytical and practical resources for sustaining the relevance and educational potency of debriefing, supporting not only learner development but also educator growth and the ongoing improvement of debriefing practice.

## Data Availability

Not applicable. No datasets were generated or for this paper.

## Footnotes

1 The Healthcare Simulation Dictionary (3rd edition) (2024), based on the APA, defines the “frame” as “a set of parameters defining either a particular mental schema or the wider cognitive structure by which an individual perceives and evaluates the world” (American Psychological Association Dictionary of Psychology, 2007, np). Lioce L, Lopreiato J. Healthcare Simulation Dictionary–Third Edition. Rockville, MD: Agency for Healthcare Research and Quality; 2024.

2 In this manuscript, terms such as *implicit*, *tacit*, or *preconceptual* are used to refer to meanings that are not yet fully formulated or made explicit, rather than to separate levels of consciousness. This usage is descriptive, pointing to different degrees of clarity or articulation of experience as it unfolds in interaction, without assuming a strict division between the conscious and the unconscious.

3 A *non-linear phenomenon* is a dynamic process characterized by non-proportional relationships among variables, recursive feedbacks, and sensitivity to initial conditions—meaning that small differences at the start are amplified over time and can lead to very different outcomes—, where the system’s evolution cannot be predicted through linear sequences or attributed to unidirectional causes, and where small perturbations can generate disproportionate and emergent effects.

4 Phenomenology is a philosophical movement, founded by Edmund Husserl, that studies consciousness and phenomena as they directly present themselves to subjective experience. It is also a research method in the social and health sciences that analyzes lived experience to understand the meaning of human experiences from a first-person perspective, exploring how individuals perceive and construct their own reality.

5 Dynamic coupling is understood, in continuity with Maturana’s concept of structural coupling, as the reciprocal process through which the organism and its environment mutually co-modulate, so that perception, conception, and conceptualization unfold simultaneously and in a correlated manner in interaction, giving rise to action and meaning in real time, without mediation by previously stored representations.

6 Nobel Prize in Medicine in 1966.

7 This statement applies equally well to the relationship between frame, action, and result when students carry out the debriefing process.

8 Intra-subjective and intersubjective dynamics refer to the internal processes through which individuals make sense of their experience, and the relational processes through which meanings are negotiated, co-constructed and transformed in interaction with others during debriefing.

9 Semiosis is the process of production and interpretation of signs carried out by living beings. Semiotics studies the structures of meaning and systems of signs that unfold in social life.

10 Juri Lotman (1922–1993), Russian-Estonian semiotician, introduced the notion of semiosphere, a concept that he elaborated by analogy to the biosphere, but composed of all possible semiotic processes (63). The semiosphere is made up of different elements, such as language, signs, codes, and cultural norms. These elements interact to determine the way meaning is produced and interpreted in a given culture. The semiosphere presents a high internal diversity of meanings in constant interaction and evolution.

11 In the framework of microgenetic theory, form can be understood as a mental configuration that emerges dynamically during the process of constituting a mental act, such as perception, thought, or the acquisition of a concept. This approach holds that every conscious mental act results from a sequence of internal organization that unfolds over time, from deep and implicit structures toward more differentiated and conscious configurations.

12 This logical system, although coming from a different theoretical framework, allows the frame-action-result relationship to be explained and understood in an emergent and non-linear way.
