# Correction: Rethinking real-time learning in debriefing: liminality, microgenesis, and semiotic borders

**DOI:** 10.1186/s41077-026-00473-4

**Published:** 2026-07-24

**Authors:** José Luis Medina, Gabriel Hervas, Isaac Calduch, Patricia Silva, Esther León

**Affiliations:** 1 Department of Didactics and Educational Organization, Faculty of Education, University of Barcelona, Barcelona, Spain; 2 Clinical Simulation Laboratory, Faculty of Medicine, University of Barcelona, Barcelona, Spain

**Correction: Adv Simul 11, 32** **(2026)**

**https://doi.org/10.1186/s41077-026-00436-9**

When this article was published, the name of the 3rd author was erroneously given as "Isaach Calduch" though it is correctly "Isaac Calduch".

The Original Article has been corrected

